# Comparative analyses of long non-coding RNA profiles in vivo in cystic fibrosis lung airway and parenchyma tissues

**DOI:** 10.1186/s12931-019-1259-8

**Published:** 2019-12-16

**Authors:** Parameet Kumar, Chaitali Sen, Kathryn Peters, Raymond A. Frizzell, Roopa Biswas

**Affiliations:** 1 Department of Anatomy, Physiology and Genetics, School of Medicine, Uniformed Services University of the Health Sciences, Room B4024, 4301 Jones Bridge Road, Bethesda, MD 20814 USA; 2 Departments of Pediatrics and Cell Biology, School of Medicine, University of Pittsburgh, Pittsburgh, PA 15261 USA

**Keywords:** Cystic fibrosis, F508del-CFTR, long non-coding RNA, mRNA, lung disease

## Abstract

**Background:**

Recent advances in the functional analyses of endogenous non-coding RNA (ncRNA) molecules, including long non-coding RNAs (LncRNAs), have provided a new perspective on the crucial roles of RNA in gene regulation. Consequently, LncRNA deregulation is a key factor in various diseases, including pulmonary disorders like Cystic Fibrosis (CF). CF is the most common life limiting recessive disease in the U.S., and is due to mutations in the CFTR gene. CF mutations, of which the most common is F508del-CFTR, prevents correct folding, trafficking and function of the mutant CFTR protein and is further manifested by the hyper-expression of pro-inflammatory cytokines and chemokines into the airway lumen leading to bronchiectasis and culminating in lung destruction.

**Methods:**

Here we report a distinct LncRNA signature and corresponding mRNAs that distinguishes CF lung (airway and parenchyma) tissues from matched non-CF controls (*n* = 4 each group), generated by microarray specific for LncRNAs which includes corresponding mRNA expressions. In silico analyses of the cellular processes that are impacted by these LncRNAs was performed using Gene Ontology (GO). A selected subset of LncRNAs were validated by quantitative real-time PCR.

**Results:**

We have identified 636 LncRNAs differentially expressed in CF airway epithelium and 1974 in CF lung parenchyma compared to matched non-CF controls (fold change ≥2, *p* < 0.05), majority of which (> 50%) are intergenic. Interestingly, 15 of these differentially expressed LncRNAs and 9 coding mRNAs are common to airway and parenchyma tissues. GO analyses indicates that signaling pathways and cell membrane functions are significantly affected by the alteration in LncRNA expressions in CF lung tissues. Seven of the differentially expressed LncRNAs, exhibit similar expression trends in CFBE41o- compared to control cells.

**Conclusion:**

Understanding the mechanisms by which these LncRNAs regulate CF disease phenotype will help develop novel therapeutic targets for CF and related pulmonary diseases, such as COPD and Asthma.

## Introduction

Cystic Fibrosis (CF) is the most common life limiting recessive disease in the U.S. and is due to mutations in the CFTR gene. CF mutations, the most common of which is F508del-CFTR, cause a massive pro-inflammatory phenotype in the lung arising from dys-regulated expression of inflammatory genes. Recently, endogenous non-coding RNA (ncRNA) molecules, including long non-coding RNAs (LncRNAs), have emerged as important therapeutic targets at the frontier of biomedical research. These LncRNAs coordinate with epigenetic factors to play a crucial role in the regulation of biological processes as well as in diseases. LncRNAs have recently emerged as novel epigenetic regulators of gene expression, including inflammatory genes. The recent FANTOM Atlas [1] has shown that many LncRNAs come from upstream enhancer elements and are functional.

LncRNAs are defined as non-coding RNAs (ncRNAs) that are transcribed by RNA polymerase II, and are at least 200 nucleotides in length [2]. LncRNAs do not have the ability to encode proteins, and they include all ncRNAs longer than 200 nucleotides (except rRNA and tRNA). These RNA molecules have provided a new perspective on the roles of RNAs in gene regulation [3, 4]. LncRNAs mostly originate within a 2-kb region surrounding the Transcription Start Site (TSS) of protein-coding genes and some originate from more distal (> 2 kb) unannotated regions. Thus LncRNAs are classified according to their position relative to protein-coding genes, and are divided into five classes [5, 6]: (i) intronic LncRNAs are located within an intron of a protein-coding gene in either direction; (ii) long intergenic ncRNAs (LincRNA) are separated by transcriptional units from protein-coding genes; (iii) bidirectional LncRNAs are transcribed in opposite directions in relation to the promoter of a protein-coding gene; (iv) antisense LncRNAs are transcribed across the exons of protein-coding genes from the opposite direction; and (v) transcribed pseudogene LncRNAs are transcribed from a gene without the ability to produce a protein.

Some LncRNAs are preferentially expressed in immune cells and play important roles in immune cell development [7]. LncRNAs are considered to be more species-, tissue- and developmental stage-specific than mRNAs [8]. Several studies have demonstrated that LncRNA deregulation has a role in various diseases [9, 10], including pulmonary disorders [11, 12]. One study indicates the role of LncRNAs in the regulation of mutant CFTR and its impact on the CF disease phenotype [13]. Suppression of LncRNA in intron 11 of CFTR, called BGAS (BG213071), or repression of its protein binding partners, has been shown to induce a 4-fold increase in mutant CFTR at the cell surface [13]. LncRNA expressions have been analyzed in CF bronchial epithelium [14] and in primary CF bronchial epithelial cells infected with *Pseudomonas aeruginosa* [15].

Here we have identified LncRNAs that are differentially expressed in lung airway and parenchyma tissues isolated from CF patients undergoing lung transplant compared to matched non-CF control tissues. Further analysis of the expression of selected subset of LncRNAs was performed by TaqMan-based qPCR assays. Seven LncRNAs exhibit similar expression trend in the CF epithelial cell line, CFBE41o-. The CF disease-specific LncRNA signature includes > 50% intergenic LncRNAs. Bioinformatic analyses of these differentially expressed CF-specific LncRNAs indicate their impact on CF-relevant cellular and biological processes, including cell membrane function as well as signaling pathways. Collectively, these LncRNAs are likely to provide new insights into epigenetic mechanisms that regulate CF disease phenotype. Understanding the associated mechanisms will ultimately help identify novel therapeutic targets for CF and related pulmonary disorders.

## Materials and methods

### Reagents

The following reagents were used: α-MEM (Sigma, M2279), 0.25% Trypsin-EDTA (Sigma, T3924), Fetal Bovine Serum (Millipore, ES-009-B), L-Glutamine (Millipore, TMS-002-C), Penicillin-Streptomycin solution (Millipore, TMS-AB2-C), Hygromycin B (Sigma, H0654-500MG) and miRVana kit (Ambion, AM1560).

### Study populations and human specimens

Lung tissues from eight subjects undergoing lung transplant were obtained for this study from the University of Pittsburgh Cystic Fibrosis Research Center: 4 CF and 4 matched non-CFs, with mean ages of 29 ± 3.8 yr and 24.4 ± 6.4 yr, respectively. Bronchial epithelial and parenchyma tissues were collected from each of these individuals, under a University of Pittsburgh Institutional Review Board approved protocol.

The procedure for collection of bronchial epithelial and parenchymal tissues was similar to that described [16]. Following lung resection, the bronchial tree from the second to sixth generation was dissected and rinsed for 24 h at 4 °C in Eagle’s MEM/HEPES on a rocker to remove any blood and mucus. After 36–48 h exposure to 0.1% protease XIV and 1% DNase in EMEM/HEPES, bronchial epithelial samples were collected by gently scraping the luminal surface using a surgical blade. Samples were then treated with Accutase and passed through a 0.22um cell filter. Microscopic evaluation of these samples revealed that this approach isolates a pure population of bronchial epithelia cells without contamination from the underlying lamina propria, which was intact following this procedure. Distal lung parenchyma samples were obtained from a lobe showing no obvious pathology or inflammation. Samples collected in this manner were snap frozen in liquid nitrogen and stored at -80 °C until RNA extraction.

### RNA profiling and statistical analyses of data

LncRNAs and mRNA profiling studies were performed by Arraystar, Inc. (Rockville, MD, USA) on RNA isolated from lung tissues. Total RNA from each sample was quantified by the NanoDrop Spectrophotometer ND-1000, and RNA integrity was assessed by agarose gel electrophoresis as well as by bioanalyzer (as part of Arraystar services). Agilent Feature Extraction software (version 11.0.1.1) was used to analyze the acquired array data. Quantile normalization and subsequent data processing were performed using the GeneSpring GX v12.1 software package (Agilent Technologies). Differentially expressed LncRNAs and mRNAs with statistical significance were identified through Fold Change filtering between two sample groups. GO analysis was applied to determine the roles that these differentially expressed mRNAs played in the identified biological pathways. Finally, Hierarchical Clustering was performed to show the distinguishable LncRNA and mRNA expression patterns among samples.

### Cell culture

CFBE41o- (Millipore, SCC160) and CFBE41o-6.2WT-CFTR (Millipore, SCC151) cells, obtained from Millipore Sigma were used for these studies. Both the cell lines were grown in Collagen I coated T75 flask (Thermo Scientific, 132,707) in minimum Eagle’s medium and supplemented with 10% Fetal Bovine Serum, 2 mM L-glutamine, 1X Penicillin-Streptomycin and 300 μg/ml Hygromycin B (CFBE41o-6.2WT-CFTR). All cells were maintained in a humidified 5% CO_2_ incubator at 37 °C.

### RT-PCR and LncRNA assays

Total RNA was isolated using the mirVana miRNA Isolation Kit, following the manufacturer’s instructions. After RNA extraction, RNA samples were reverse transcribed by the High Capacity cDNA Reverse Transcription Kit (Applied Biosystems, 4,368,813). Real-time quantification of individual LncRNAs in CF cell lines was performed with specific TaqMan assays designed using the custom TaqMan assay design tool available from Thermo Fisher. Real-time PCR data were normalized to the endogenous β-actin control. The relative fold changes of LncRNAs were analyzed using the *2*^-ΔΔ*CT*^ *method.*

### Statistical analysis

Statistical analysis was performed using Excel. Significance values (*p* ≤ 0.05) were determined by student’s t-test. Error bars on graphs represent SEM.

## Results

### LncRNA expression profiles in CF lung airway and parenchyma tissues

**Fig. 1:**
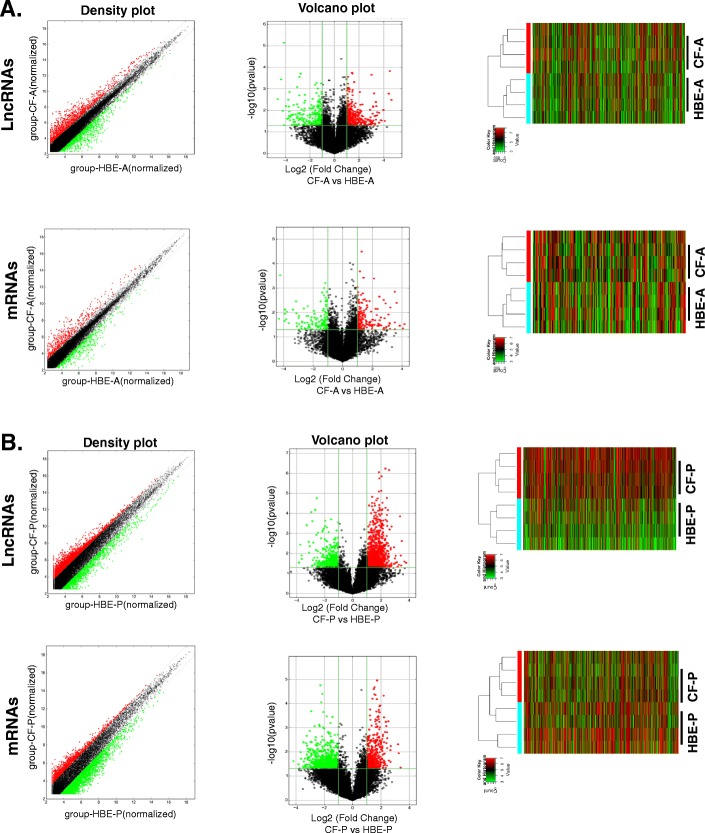
Analyses of LncRNA in CF lung tissues**.** The expressions of LncRNA and corresponding mRNAs significantly (*p* < 0.05) altered in CF lung tissues compared to matched non-CF control tissues (*n* = 4 each) were analyzed by LncRNA arrays: **a** airway and **b** parenchyma tissues. Density plots depict the variation in expression: X and Y axes represent averaged normalized values in each group (log2). Volcano plots depict the fold-changes: each dot represents a single LncRNA or mRNA and is colored black unless it was differentially expressed. Hierarchical clustering and the heat map indicate the expression level of the transcripts significantly altered in CF tissues compared to non-CF controls: red represents increased expression, while green represents reduced expression. (CF-A, CF lung airway tissue; HBE-A, control airway tissue; CF-P, CF lung parenchyma tissue; HBE-P, control lung parenchyma)

To understand the role of LncRNAs in CF lung disease we performed comprehensive analyses of LncRNAs and corresponding mRNA expression profiles in lung tissues, both airway and parenchyma, obtained from CF patients undergoing lung transplant. These data were compared with those obtained from age (ranging from 23 to 36 years) and sex-matched (4 male and 4 female) healthy controls (Table 1). The expression of LncRNAs and mRNAs were analyzed by microarray (Human LncRNA Array v3.0, Arraystar, Inc.), which is comprised of ~ 40,173 LncRNAs and ~ 20,730 mRNAs. We have identified 636 differentially expressed LncRNAs in CF airway tissues (Fig. 1a) and 1974 differentially expressed LncRNAs in CF lung parenchyma tissues (Fig. 1b) compared to matched respective healthy controls (*n* = 4 each group, fold change ≥2, *p* < 0.05). As depicted in Fig. 1, when all these LncRNAs are compared using a hierarchical clustering algorithm, the dendrogram clearly distinguishes CF tissues from matched controls. The corresponding mRNA expression profiles also clearly distinguish CF tissues from controls (Fig. 1a and b).

**Table 1:** Patient lung tissue data

Sample	CFTR	Age	Gender
CF1	F508del/ F508del	32	M
HBE1	WT	34	M
CF2	F508del/ F508del	32	F
HBE2	WT	36	F
CF3	F508del/ F508del	28	F
HBE3	WT	25	F
CF4	F508del/ F508del	24	M
HBE4	WT	23	M

The description of CF and non-CF control tissue samples used, including age and gender, are indicated

**Fig. 2:**
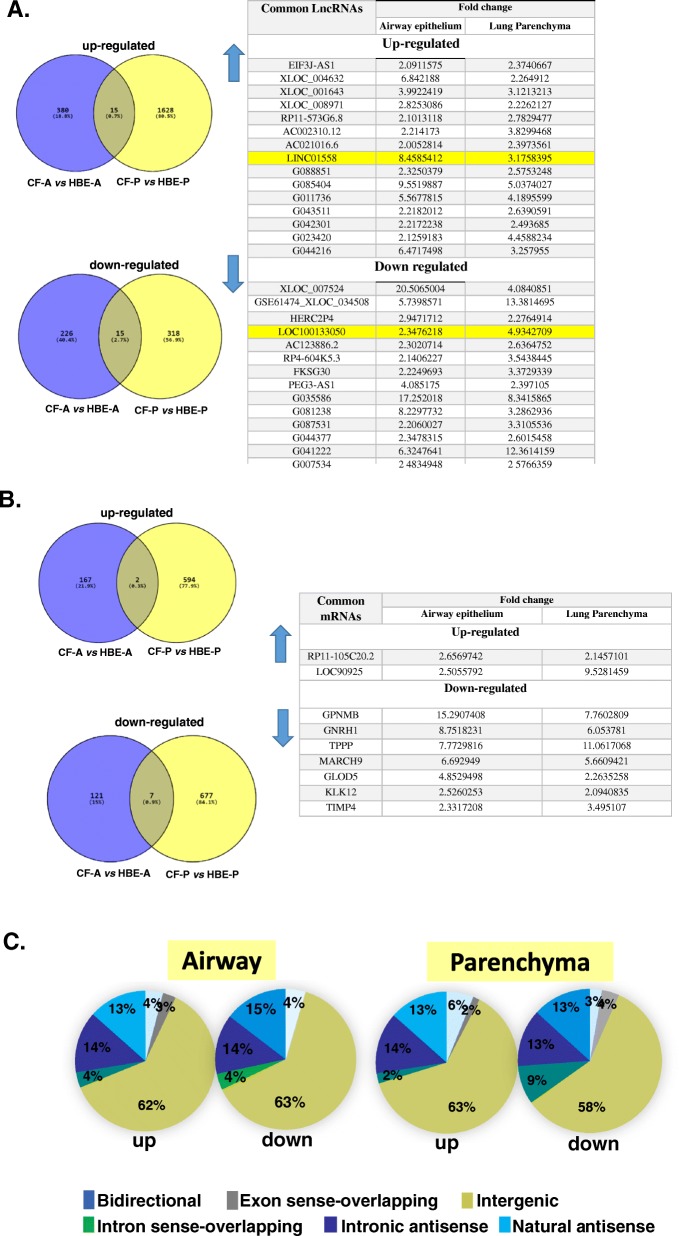
Comparative analyses and classification of LncRNAs significantly altered in CF airway and parenchyma tissues. Venn diagrams indicate the differentially expressed (**a**) LncRNAs and **b** mRNAs that are common to airway and parenchyma tissues: up-regulated and down-regulated in CF compared to control tissues. The LncRNAs and mRNAs that exhibit similar expression pattern in the CF airway and parenchyma tissues are listed in the adjacent tables. The two LncRNAs that were validated in cell line models are highlighted. **c** The pie charts indicate the classification of the aberrantly expressed LncRNAs. The intergenic LncRNAs account for majority (58–63%) of the differences in CF airway as well as lung parenchyma tissues compared to non-CF control tissues

Interestingly, 15 LncRNAs exhibit similar expression trends in CF lung airways as well as in CF parenchyma tissues (Fig. 2). The Venn diagrams **(**Fig. 2a) indicate that of the total number of up-regulated LncRNAs in CF tissues, 15 LncRNAs (0.7%) are common to both CF airway and parenchyma tissues, while 380 LncRNAs (18.8%) are unique to CF airway and 1628 (80.5%) are unique to CF parenchyma tissues compared to matched controls. Similarly, amongst those LncRNAs that are down-regulated in CF tissues; coincidentally, 15 LncRNAs (2.7%) are common to both CF airway and parenchyma tissues, while 226 LncRNAs (40.4%) are unique to CF airway and 318 (56.9%) are unique to CF parenchyma tissues compared to matched controls. Additionally, the analyses of corresponding mRNAs in these tissues indicate that two mRNAs are up-regulated and seven mRNAs are down-regulated both in airway and parenchyma tissues (Fig. 2b).

### Classification of the differentially expressed LncRNAs in CF lung tissues

Analyses of the differentially expressed LncRNAs indicate that majority of the differentially expressed LncRNAs belong to the class of intergenic LncRNAs. The classification type as well as respective fold changes of the LncRNAs that are significantly up-regulated or down-regulated in CF tissues are listed in Tables 2 and 3. Of the top 10 LncRNAs that are significantly up- or down-regulated in the CF airway tissues, EEF1E1-BLOC1S5 and ARHGAP19-SLIT1 are read through transcripts, uc.363+, uc.8- and uc.476 are ultra-conserved LncRNAs, while the remaining have not been characterized. A few of the top 10 LncRNAs significantly altered in the CF parenchyma tissues have been characterized, and include the two up-regulated natural anti-sense non-coding transcripts, MIR3617 and POT1-AS1, and two intergenic LncRNAs, LINC00176 and LINC01023.

**Table 2:** LncRNA expressions in CF airway tissues

Up-Regulated LncRNAs	Fold Change↑	*P*-value	Type	RNA length
G065529	24.7458154	0.003257887	intergenic	613
uc.363+	22.6910199	0.000149697	intergenic	265
EEF1E1-BLOC1S5	21.3884736	0.001680515	exon sense-overlapping	2992
RN7SKP237^a^	16.9873196	0.03106286	intronic antisense	301
ARHGAP19-SLIT1^a^	15.7966699	0.00962748	exon sense-overlapping	1916
G063528	14.394274	0.029592867	intergenic	741
RP1-283E3.4	13.4799713	0.028579511	intronic antisense	1808
GSE61474_XLOC_052517	12.3432679	0.037357762	intergenic	921
G069001	12.2990892	0.012996569	intergenic	456
uc.8-	11.0711707	0.012121961	intron sense-overlapping	216
Down-Regulated LncRNAs	Fold Change↓	*P*-value	Type	RNA length
G023050	24.8676423	0.002996172	intergenic	4752
XLOC_007524	20.5065004	0.000367446	intergenic	306
RP4-663 N10.1	18.7038359	0.010580021	intronic antisense	2610
G035586	17.252018	7.27187E-06	intergenic	3265
LOC101928663	17.1118104	0.049196654	intergenic	1073
G033946	15.5916483	0.006354509	intronic antisense	2310
AC003092.1^a^	14.6286547	0.02466049	intergenic	639
LOC101928516^a^	13.661028	0.003223776	intergenic	677
G043932	13.5022191	0.023524085	intergenic	4908
uc.476	13.2472716	0.006668107	intergenic	238

The LncRNAs that are significantly up-regulated and down-regulated in CF airways tissues compared to non-CF controls are listed. (^a^Validated by qPCR)

**Table 3:** LncRNA expressions in CF parenchyma tissues

Up-Regulated LncRNAs	Fold Change↑	*P*-value	Type	RNA length
MIR3615	13.9421449	0.028800825	natural antisense	565
XLOC_009603	12.7909929	0.025368402	intergenic	873
G083378	11.2370894	0.002389514	intergenic	763
RP11-466A19.8	10.8534252	0.03983941	intron sense-overlapping	927
LINC00176	10.6662478	0.012646718	intronic antisense	5264
G012592	10.3110888	0.000140675	intergenic	1050
G018678	9.6891061	0.036809781	intergenic	1310
G044675	9.1156866	0.045874645	intergenic	1071
POT1-AS1	8.7808728	0.027374905	natural antisense	3983
XLOC_006737	8.2777265	0.012895875	intergenic	983
Down-regulated LncRNAs	Fold Change↓	*P*-value	Type	RNA length
G065252	14.331561	0.028187016	intergenic	1056
GSE61474_XLOC_034508	13.3814695	0.003626576	intergenic	1565
LOC101928697	12.5462712	0.012533081	bidirectional	574
G041222	12.3614159	0.018597696	intergenic	618
AC099850.1	9.7851638	0.000389157	intronic antisense	1338
LINC01023^a^	8.861641	0.002639581	intergenic	457
G027019	8.6429315	0.009020488	intergenic	777
G035586	8.3415865	0.004331082	intergenic	3265
RP11-667 M19.2	7.7994756	8.64985E-05	intron sense-overlapping	586
G087905	7.5150766	0.042188797	intergenic	1443

The LncRNAs that are significantly up-regulated and down-regulated in CF parenchyma tissues compared to non-CF controls are listed. (^a^Validated by qPCR)

**Fig. 3:**
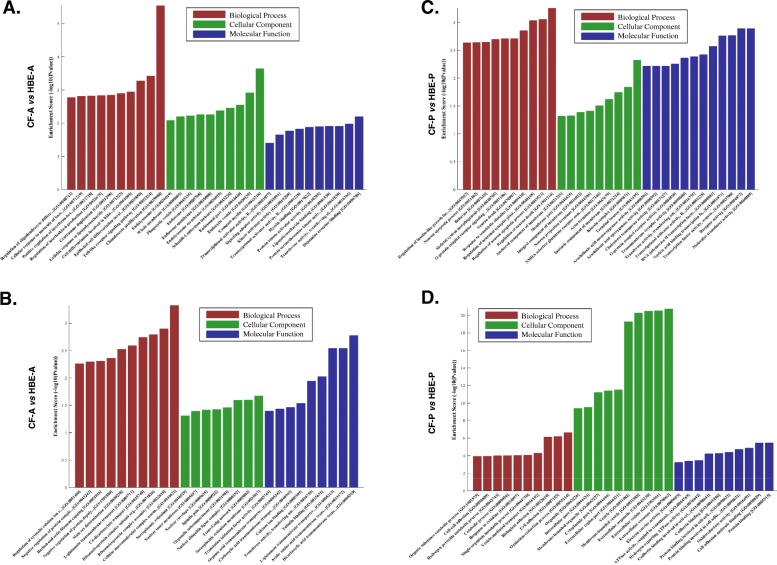
Analyses of cellular processes affected by differentially expressed LncRNAs in CF lung airway and parenchyma tissues. Gene Ontology (GO) analyses was used to evaluate the impact of the aberrantly altered LncRNAs in (**a**) up-regulated in CF airway, **b** down-regulated in CF airway, **c** up-regulated in CF lung parenchyma, and **d** down-regulated in CF lung parenchyma tissues compared to respective non-CF controls. The significantly impacted Biological Processes, Cellular Component and Molecular Functions are depicted by corresponding histograms that indicate the enrichment score

As depicted by the Venn diagram (Fig. 3a), the intergenic LncRNAs account for the majority (58–63%) of the differences in CF airway as well as CF lung parenchyma tissues compared to respective control tissues. LncRNAs that are in the natural antisense and intronic-antisense categories accounted for about 13–15% of the total, while the remaining categories that include exon-sense overlapping, intron-sense overlapping and bi-directional accounted for < 10% of the total LncRNAs.

### Analyses of LncRNA-mRNA networks

In silico analyses of the differentially expressed LncRNAs and mRNAs networks were performed with Gene Ontology (GO) analyses program. Our data indicate that CF relevant biological processes are associated with signaling pathways; cellular components that include the intracellular vesicle, and membrane systems; and molecular functions including transcriptional activator activity are impacted in CF lung tissues compared to matched control tissues. Consistent with the CF disease phenotype, the array data indicate cell membrane function, including ion transport, is significantly affected by the aberrant expression of LncRNAs in CF lung tissues (Fig. 3a-d).

The Biological Processes (BP) that are significantly up-regulated in CF airway tissues, include regulation of inflammatory molecules (viz., IL-6, Interferon-beta), response to LPS as well as Toll-like Receptor (TLR) signaling. Those BP that are down-regulated significantly in CF airways, include regulation of cytosolic calcium ion, membrane transport functions. The two key BP that are up-regulated in CF parenchyma tissues include regulation of Insulin-like Growth Factor signaling and G-protein couple receptor signaling. Moreover, cell-cell adhesion, response to cytokine, vesicle-mediated transport, oxidation-reduction reactions are reduced in the CF parenchyma tissues. The overall analyses of the Cellular Component (CC) on CF airways and parenchyma tissues indicate significant impact on membrane structure, vesicles, organelles (both intracellular and extracellular), exosomes, and trans-golgi network. Consistently, the overall Molecular Function (MF) impacted in the CF lung tissues include ion transport, membrane transport activity, ligand binding activity (viz. LPS), membrane receptor activity (viz. G-protein receptor binding).

### Validation of selected LncRNAs in CF epithelial cells

**Fig. 4:**
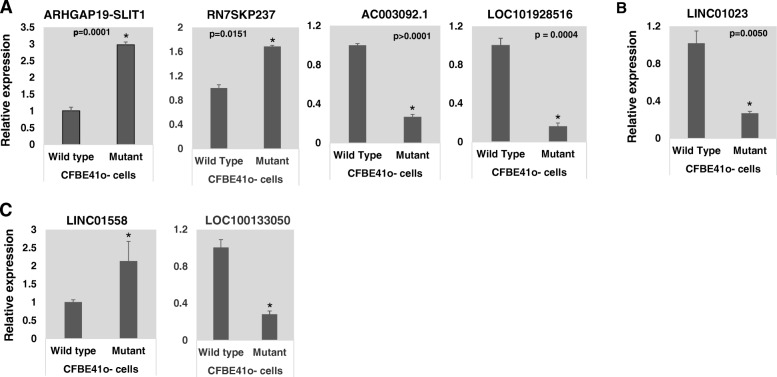
Validation of LncRNAs**.** The levels of LncRNAs differentially expressed in (**a**) CF airway tissues, **b** CF parenchyma tissues and **c** those that exhibit similar trend in both CF airway and parenchyma tissues were analyzed (by TaqMan qPCR assays) in F508del-CFTR CF cell line CFBE410-, and in control cell line containing WT-CFTR

Subsequent to comprehensive analyses of LncRNAs in CF lung tissues, we validated a selected subset of LncRNAs in cell culture models. We thus performed quantitative real-time PCR-based validation of selected LncRNAs in F508del-CFTR CF airway epithelial cell line, CFBE41o-, compared to WT-CFTR control cell line. Seven of the LncRNAs that are differentially expressed in CF lung tissues compared to matched non-CF control tissues exhibit similar expression trends in CF cell lines (Fig. 4). These include RN7SKP237 and ARHGAP19-SLIT, that are up-regulated, and AC003092.1and LOC101928516 that are down-regulated, in CF airway tissues (Fig. 4a); LINC01023 which is down-regulated in CF lung parenchyma tissues (Fig. 4b); and two that are common to CF airway and parenchyma tissues, LINC01558 is up-regulated, and LOC100133050 is down-regulated (Fig. 4**c**). The data indicate that the expression levels of all these LncRNAs were significantly (*p* < 0.05) different between the CF bronchial epithelial cell line, CFBE41o- as compared to that in control cells. Further studies are focused on functional analyses of these LncRNAs.

## Discussion

LncRNAs have emerged as key regulators of cellular processes and have been found to be aberrantly expressed in various diseases. The role of LncRNAs in CF has not been studied extensively. Here we report a LncRNA signature that distinguishes F508del-CFTR CF lung airway and parenchyma tissues from matched controls (*n* = 4 each group). The CF airway tissues have 636 differentially expressed LncRNAs, while the CF lung parenchyma tissues exhibited 1974 differentially expressed LncRNAs, compared to respective matched control tissues. The majority (> 50%) of these LncRNAs belong to the class of intergenic LncRNAs (LincRNAs). Moreover, 15 of these LncRNAs are elevated in both types of CF lung tissues, airway as well as parenchyma tissues, and coincidentally 15 LncRNAs also exhibit reduced expression in both types of CF tissues. Interestingly, we also find mRNAs that exhibit a similar trend in the CF airway and parenchyma tissues. Though we were able to analyze a limited number of clinical samples (*n* = 4 per group), we find a distinct LncRNA signature for CF airway and parenchyma tissues.

Comprehensive analyses of LncRNAs in CF tissues have identified two read through non-coding transcripts, EEF1E1-BLOC1S5 and ARHGAP19-SLIT1, an antisense LncRNA RN7SKP237, and 3 ultra-conserved LncRNAs, uc.363+, uc.8- and uc.476, that are significantly up- or down-regulated in the CF airway tissues. While in the CF parenchyma tissues two natural anti-sense non-coding transcripts, MIR3617 and POT1-AS1, are up-regulated, and two intergenic LncRNAs, LINC00176 and LINC01023 exhibit opposite trend. While LINC00176 is a myc-target and is up-regulated, LINC01023 is known to be associated with IGF1R/Akt pathway and is down-regulated in the CF lung parenchyma tissues. The other significantly altered LncRNAs (see Tables 2 and 3) have not been characterized. To date there have been two reports directly focused on determining the expression of LncRNAs in CF [14, 15]. Both of these studies have identified LncRNA expression profiles in primary CF cells type different from those used in our study. McKiernan et al., [14] analyzed LncRNAs in cells isolated from bronchial brushings of CF patients and non-CF controls and have identified XIST and TLR8 to be differentially expressed in these samples. However, we did not find significant alteration in the expression of these LncRNAs in the CF lung tissues, which were isolated from CF patients undergoing lung transplant. The source of the samples could be contributing to these differences. Moreover, the study by Balloy et al. [15] also used different cell types, primary CF bronchial epithelial cells which were infected with *Pseudomonas aeruginosa,* and have identified the differential expression of distinct non-coding transcripts.

Analyses of the corresponding coding mRNA expressions yields interesting data. There is a total of 9 mRNAs which are commonly altered in expression in the CF airway and parenchyma tissues (Fig. 2b). The proteins coded by the two up-regulated mRNAs, RP11-105C20.2 and LOC90925, have not been characterized. However, the 7 mRNA transcripts that are down-regulated in the CF lung tissues (both airway and parenchyma) are interestingly associated with CF disease phenotype. GPNMB levels have been reported to be markedly increased in serum and circulating leukocytes from CF patients compared to healthy controls [17]. On the contrary we find decreased levels of the GPNMB transcript in CF lung tissues. Also, interestingly CFTR has been reported to regulate GnRH secretion and thereby regulate sexual maturation and infertility in CF women [18]. MARCH9, an E3 ubiquitin ligase, is down-regulated in both CF airway and parenchyma tissues. Its activity is similar to that of MARCH2, which promotes ubiquitination and subsequent lysosomal degradation of mature CFTR [19]. TPPP has been shown to reduce microtubule formation rates and decrease microtubule acetylation, replicating the CF cellular condition [20]. GLOD5 is predicted to interact with HDAC6, and interestingly depletion of HDAC6 has been shown to rescue CF disease phenotype in a CF mouse model [21]. KLK12 expression has been shown to be suppressed in CF cells compared to control cells 2 h post-infection with *Pseudomonas aeruginosa* [15]. TIMP4 restricts fibrosis by regulating ECM deposition (through TGF-beta signaling pathway) and also causes restriction of inflammation [22]. Consistently, TIMP4 expression is reduced in CF lung tissues.

In silico analyses of these differentially expressed LncRNAs and mRNAs by GO indicates impact on relevant CF disease-specific pathways and cellular processes. The biological processes impacted include Toll-like receptor signaling, oxidation-reduction process, regulation of IL-6 production, regulation of cytosolic Calcium ions, G-protein signaling, etc. Consistently, cellular processes and molecular functions impacted include, vesicle transport, membrane function, LPS-binding, cell adhesion, oxidoreductase activity, transcriptional and translation activity, etc. These bioinformatic analyses support the importance of the array data.

Subsequently, we analyzed the expression of the top 10 up-regulated and top-10 down-regulated LncRNAs in CF airway tissues compared to control tissues (see Table 2) in CF airway epithelial cell line using TaqMan assay specific for each of these LncRNAs. Due to lack of sufficient RNA samples from CF tissues and also since in vitro cell culture systems will be employed for further mechanistic studies, we have analyzed the expression of selected LncRNAs in CFBE41o- cell lines as well as in respective control cell line with WT-CFTR. The LncRNAs which exhibit similar expression trends in CF epithelial cell lines as compared with those observed in the microarray data obtained from CF lung tissues, include RN7SKP237, ARHGAP19-SLIT1, AC003092.1 and LOC101928516 differentially expressed in the CF airway tissues; LINC01023 which is down-regulated in CF lung parenchyma tissues; and LINC01558 and LOC100133050, that exhibit similar expression trend both in the CF airway and parenchyma tissues.

The biological functions of the LncRNAs, RN7SKP237 and ARHGAP19-SLIT1, are poorly understood. RN7SKP237 is a pseudogene, which belongs to the anti-sense family of LncRNAs, and is associated with the microsomal glutathione transferase gene (MGST2). Interestingly, MGST2 protein catalyzes the biogenesis of leukotriene C4, which is a potent pro-inflammatory mediator of the pathophysiology of CF [23]. ARHGAP19-SLIT1 is a read through transcript between neighboring Rho-GTPase-activating protein 19 and slit homolog1 (SLIT1) and is a target of non-sense-mediated decay (NMD). This LncRNA spans two genes, ARHGAP19 and SLIT1. Interestingly, the coding transcript ARHGAP19 is a negative regulator of Rho GTPases, which are involved in cell migration, proliferation, and differentiation, actin remodeling, and G1 cell cycle progression [24], again relevant to CF disease. AC003092.1 has been shown to induce apoptosis in Glioblastoma (GB) by increased sensitivity to chemotherapy and thereby a potential therapeutic target for GB [25]. LINC01023 is a regulator of the IGF1R/Akt pathway in glioma [26], again a signaling pathway that has been shown to regulate CF lung disease. LOC101928516, LINC01558 and LOC100133050 have not been characterized.

The differentially expressed LncRNAs in CF may play important roles in the pathophysiology of CF lung disease. The LncRNA expression profile and the corresponding mRNA expression implicate CF relevant biological processes as affected in the CF disease tissues compared to control tissues. Further studies are being directed towards understanding the role of these LncRNAs in the disease phenotype of CF, including lung inflammation and mutant CFTR function. These mechanisms will serve as paradigms for similar complex processes and are expected to lead to the development of novel therapeutic targets in CF and other pulmonary disorders, such as COPD and Asthma.

## Data Availability

The datasets generated and/or analyzed during the current study are not publicly available due [Ongoing research with these] but are available from the corresponding author on reasonable request.

## Abbreviations

CF: Cystic Fibrosis
CFTR: Cystic Fibrosis Transmembrane Conductance Regulator
GO: Gene Ontology
LincRNA: long intergenic non-coding RNA
LncRNA: long non-coding RNA
